# Effect of *Eucommia ulmoides* leaves on hyperuricemia and kidney injury induced by a high-fat/high-fructose diet in rats

**DOI:** 10.22038/ijbms.2022.62681.13867

**Published:** 2022-04

**Authors:** Man Gong, Hong Zhang, Xiaoqian Liu, Qingxia Li, Yang Zhang, Weijin Zhang, Na Huang, Anying Chen, Liping Dai, Zhimin Wang

**Affiliations:** 1 Henan University of Chinese Medicine, Henan Zhengzhou 450046, China; 2 Engineering Technology Research Center for Comprehensive Development and Utilization of Authentic Medicinal Materials from Henan, Henan Zhengzhou 450046, China; 3 The Second Affiliated Hospital of Zhengzhou University, Henan Zhengzhou,450014, China; 4 Institute of Chinese Materia Medica, China Academy of Chinese Medical Sciences, Beijing 100700, China; 5 Henan Zhongjing Key Laboratory of Prescription, Henan Zhengzhou 450046, China; # These authors contributed equally to this work

**Keywords:** Eucommia ulmoides leaves, High-fat and high-fructose diet, Hyperuricemia, Kidney injury, Molecular docking, Network pharmacology

## Abstract

**Objective(s):**

To investigate the protective and preventive treatment effects of *Eucommia ulmoides* leaves on a rat model of high-fat and high-fructose diet (HFFD) induced hyperuricemia and renal injury.

**Materials and Methods:**

Network pharmacology and molecular-docking methods were used to predict the effects and action mechanisms of the major components of *E. ulmoides* leaves on hyperuricemia. Combining literature collection, we used SciFinder and the Traditional Chinese Medicine Systems Pharmacology Database (TCMSP) and Analysis Platform to collect *E. ulmoides* leaf flavonoid and iridoid components. Swiss Target Prediction, Similarity ensemble approach (SEA), GeneCards, and the Online Mendelian Inheritance in Man (OMIM) database were used to obtain core targets, and the Search Tool for Recurring Instances of Neighbouring Genes (STRING) protein database was used as core target for gene ontology enrichment Set and Kyoto Encyclopedia of Genes and Genomes (KEGG) analyses. Molecular docking was applied to predict the pathways regulating the metabolism of uric acid. The selected targets and targeting efficacy were validated using a rat model of hyperuricemia and renal injury induced by a high-fat and high-fructose diet.

**Results:**

A total of 32 chemical components with effective targets, which regulated the PI3K-AKT pathway and endocrine resistance, were collected. Molecular docking results showed that iridoids and flavonoids are bound to proteins related to inflammation and uric acid metabolism. In addition, it was verified via animal experiments that an *E. ulmoides* leaf extract ameliorated hyperuricemia, renal injury, and inflammation, which are closely related to the targets Interleukin- 6 (IL-6), Tumor necrosis factor-α (TNF-α), Toll-Like Receptor 4 (TLR4), and Glucose transporter 9 (GLUT9).

**Conclusion:**

*E. ulmoides* leaf flavonoids and iridoids ameliorate hyperuricemia and uric-acid–induced inflammation through a multi-component, multi-target, and multi-pathway mechanism, which provides a theoretical basis for the development of therapeutics from *E. ulmoides* leaf components.

## Introduction

Uric acid (UA) is the end product of purine metabolism, and fructose metabolism produces UA salts, leading to a rapid increase in serum UA levels (1, 2). Diets highly rich in fat and fructose have been reported to be associated with increased serum UA levels (3). Hyperuricemia is a biomarker of cardiovascular morbidity and mortality (4), and UA crystals can activate NOD-like receptor thermal protein domain associated protein 3 (NLRP3) inflammasomes in various tissues, thereby triggering hyperuricemia-related inflammatory diseases, such as gout, metabolic syndrome, and kidney injury (5, 6).

*Eucommia ulmoides* Oliver is a rare tree species from China. Its bark and leaves have anti-inflammatory, anti-oxidant, and liver- and kidney-protecting effects and are used as medicines to lower blood pressure and blood lipid and sugar levels (7-11). *E. ulmoides* leaves are rich in iridoids, flavonoids, lignans, phenolic acids, steroids, triterpenes, and many other chemicals, among which the main active ingredients are rutin, hyperoside, chlorogenic acid, aucubin, quercetin, geniposide, and kaempferol (12). The ethyl-acetate extract of *E. ulmoides* leaves has been shown to reduce serum UA levels and improve kidney function in hyperuricemic rats (13). The flavonoids rutin, quercetin, and kaempferol can all reduce serum UA levels, enhance the renal excretion of UA, and improve renal function. A large number of anti-inflammatory iridoid compounds, such as aucubin and geniposide, exist in *E. ulmoides* leaves (14, 15). Accordingly, such iridoids and flavonoids are the main pharmacological components of *E. ulmoides* leaves and constitute the basis for development of anti-inflammatory and anti-hyperuricemic drugs.

At present, the treatment of hyperuricemia and UA-induced inflammation relies on non-specific drugs, which often have strong side effects. Thus, safer and more effective drugs are urgently needed, especially those based on natural products. Network pharmacology can systematically predict the action mechanisms of multi-component targets and can help further elucidate the holistic and systematic nature of Chinese medicines. Molecular-docking technology can help us understand how compounds interact with their molecular targets. In this study, network pharmacology and molecular-docking methods were used to explore the potential targets and action mechanisms of iridoids and flavonoids in the treatment of hyperuricemia and UA-induced inflammation. Additionally, a high-fat and high-fructose diet (HFFD)-induced rat model of hyperuricemia and kidney injury was used to verify the therapeutic effects of *E. ulmoides* leaves from the perspective of regulating UA metabolism, improving serum inflammation, and reversing kidney damage, thus providing a theoretical basis for the development of therapeutics based on *E. ulmoides* leaf components.

## Materials and Methods

***Materials***

*E. ulmoides* leaves (EUL) were provided by Henan Golden Eucommia Agricultural Technology Co., Ltd. (Origin: Xuchang City, Henan Province, batch number: 20200510), which was identified as *E. ulmoides* Oliver by Professor Dai Liping of Henan University of Chinese Medicine of dried leaves. Weigh 2.5 kg of *E. ulmoides* leaves, soak for 1 hr, add 10 times the amount of water, extract twice, 1.5 hr each time, filter to obtain an extract, combine the extracts, recover under reduced pressure, freeze-dry, and obtain *E. ulmoides* leaf-water-extract freeze-dried powder, 17.85%.

***Animals***

Sixty specific pathogen-free male Wistar rats (190 ± 20 g), were kept in an ambient temperature range of 22 ± 1 °C, 12 hr light/dark cycle, 50%–60% relative humidity, *ad libitum* diet and drinking water, the design of animal experiments were approved by the Ethics Committee of Experimental Animals of Henan University of Chinese Medicine. Rats were purchased from Jinan Pengyue Laboratory Animal Breeding Co., Ltd., license number: SYXK (Lu) 20190003, experimental unit license number SCXK (Henan) 2020-0004, quality inspection unit: Shandong Provincial Laboratory Animal Center.

***Construction of the database of the chemical constituents of E. ulmoides iridoids and flavonoids***

We collected and sorted out the chemical components of *E. ulmoides* iridoids and flavonoids based on literature data and databases to establish a major component library by using SciFinder (https://scifinder.cas.org/) and the Traditional Chinese Medicine Systems Pharmacology Database and Analysis Platform (TCMSP) (https://old.tcmsp-e.com/tcmsp.php) to confirm these components’ structure. The ChemOffice software was used to transform the structure of each compound into a 3D structure and minimize its energy. All data were saved in mol2 format.

***Screening of common targets of the major chemical components of E. ulmoides on hyperuricemia and UA-induced inflammation***

The Swiss Target Prediction (http://www.swisstargetprediction.ch/) and Similarity ensemble approach (SEA) (http://sea.bkslab.org/) databases were screened to search for compound-related targets. The keywords were “Hyperuricemia,” “Gouty arthritis,” and “Uric nephritis.” Additionally, for disease-related targets, the GeneCards (https://www.genecards.org/) and Online Mendelian Inheritance in Man (OMIM) (http://www.omim.org/) databases were screened.

***Construction of the compound–candidate-target interaction network***

We sorted the selected 32 compounds and related targets and then mapped the targets with those related to hyperuricemia, gouty arthritis, and UA nephritis to obtain candidate targets. Using the Cytoscape 3.6.0 software, the composition–action-target network diagram was constructed.

***Protein-protein interaction (PPI) network construction and analysis***

By using the Search Tool for Recurring Instances of Neighbouring Genes (STRING) (https://STRING-db.org/) database, the retrieved potential targets of the 32 compounds for regulating hyperuricemia were correlated, and the Organization was defined as “*Homo sapiens*” to obtain the PPI network diagram. A ≥ 0.400 confidence score of correlation was set as the cutoff value to obtain PPI results. The above-mentioned PPI results were imported into the Cytoscape 3.6.0 software for visualization and to draw the PPI network. The two indicators of Degree and Closeness Centrality were selected to be greater than the average value as the standard to screen core targets for gene ontology (GO) enrichment and KEGG analyses.

***GO analysis and enrichment analysis of the KEGG signaling pathway***

WebGestalt database (http://www.webgestalt.org/) was used to conduct GO classification enrichment analysis, and the threshold was set at FDR<0.05. KOBAS 3.0 data (http://kobas.cbi.pku.edu.cn/) were adopted, and KEGG pathway enrichment analysis was performed on the hyperuricemia-modulating candidate targets of the 32 compounds identified in* E. ulmoides* leaves. We used online tools (https://www.omicshare.com/tools/) to draw a KEGG advanced bubble chart and selected the relevant pathway with *P*<0.05.

***Molecular docking***

The ChemOffice software was used to construct the 3D structure of each compound. The generated document was saved in the *mol2 format, and the energy of the compound was minimized. The 3D structure of the target protein was downloaded from the Protein Data Bank (PDB) database (https://www.rcsb.org/). The Discovery Studio software was used to perform operations such as water removal and hydrogenation on the protein, and an effective single 3D conformation was generated by minimizing the energy of the compound. Active ingredients of the *E. ulmoides* leaf extract that had binding energy ≤ −5.0 kJ/mol were selected as the screening basis for the treatment of hyperuricemia and UA-induced inflammation. The online drawing tool -CDOCKER-INTERACTION-ENERGY served as an indicator to analyze the molecular-docking results, and the online heat-map drawing tool V2.16 (https://www.lc-bio.cn/) was used to draw the docking results. Finally, cluster analysis was performed.

***Experimental validation***

*Modeling and grouping*

Wistar rats were adaptively fed for 7 days and then randomly divided into 5 groups according to body weight, namely the normal group CON (0.5% carboxymethylcellulose), high-dose *E. ulmoides* leaf-extract group CON+EULH (200 mg/kg), model group HFFD (0.5% carboxymethylcellulose), model + low-dose* E. ulmoides* leaf-extract group HFFD+EULL (100 mg/kg), model + high-dose* E. ulmoides* leaf-extract group HFFD+EULH (200 mg/kg), and metformin group HFFD+MF (100 mg/kg) with 10 rats per group. The rats in the CON group were provided with the ordinary chow and pure water; the rats in the other groups were provided with HFFD (containing 17% fat and 17% fructose) and 20% fructose water. Drug administration by gavage was started after 8 w, and the body mass of the rats was measured weekly.

*Specimen collection*

After 8 weeks of continuous administration, the rats were anesthetized with 10% chloral hydrate, and blood was collected from the abdominal aorta. The blood specimens were placed in centrifuge tubes at room temperature (20-30 °C) for approximately 2 hr for natural clotting. After complete clotting, the blood was centrifuged at 3000 rpm/min for 5 min to separate the serum. The upper layer (serum) was taken and stored at –80 °C to determine the biochemical indices. The kidneys were quickly stripped to remove the fascia and excess fat, rinsed with cold saline, and blotted using a filter paper. The kidneys were then weighed, and the organ index was calculated. Testing of serum Uric acid (UA), creatinine (Cr), and Urea (BUN) was by Nanjing Jiancheng Bioengineering Institute, China.

*Determination of serum levels of inflammatory factors*

Enzyme-Linked Immunosorbent Assay (ELISA) was used to determine the TNF-α and IL-6, TNF-α and IL-6 ELISA Kit, (Guangzhou Darwin Biotechnology Co., Ltd, China) levels in rat serum.

*Hematoxylin-eosin (HE) staining for pathological assessment of the kidney*

The kidneys were quickly extracted from the rats, rinsed with cold saline, and fixed in 4% paraformaldehyde for 24 hr. Afterward, they were paraffin-embedded and sliced into 5 µm-thick sections. The sections were placed on glass slides, stained with HE, and then dehydrated using an ethanol gradient and cleared using xylene. Histopathological changes were observed under a microscope.

*Immunostaining for pathological assessment of the kidney*

Immunofluorescent analyses for TLR4 and GLUT9 (Servicebio, Wuhan, China) proteins were performed to observe pathological tissue changes under a fluorescent fiber microscope. After the kidneys were extracted and rinsed as described above, they were blotted with a filter paper, weighed, and then placed in 15 ml centrifuge tubes with 4% paraformaldehyde solution to be fixed for 24 hr. Paraffin sections were prepared, dewaxed, and immunofluorescence stained for TLR4 and GLUT9 proteins under a fluorescent microscope to observe the changes in pathological tissue using Image J software. The expression of TLR4 and GLUT9 proteins was analyzed.

***Statistical analysis***

The GraphPad Prism 8.0.1 software was used for statistical analysis. Each value presented corresponds to the mean ± SD from three independent experiments. One-way Analysis of Variance (ANOVA) was used for comparisons among multiple groups, and Tukey’s test was adopted for multiple comparisons. *P*<0.05 and *P*<0.01 were set as the standards for significant and extremely significant differences, respectively.

## Results

***Construction of the database of the chemical constituents of E. ulmoides iridoids and flavonoids***

Using literature data and the SciFinder and TCMSP databases, a total of 32 small-molecule compounds with clear structural information of the chemical components of *E. ulmoides* iridoids and flavonoids were obtained. They included 22 iridoids (iridoid01–22) and 10 flavonoids (flavonoid01–10), as shown in Table 1.

***Major components of E. ulmoides leaves and the common targets related to the treatment of hyperuricemia and UA-induced inflammation***

**Figure 1:**
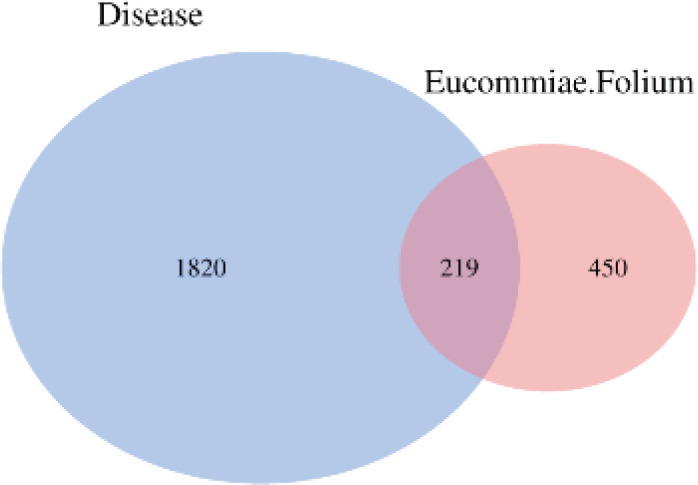
Common targets related to hyperuricemia and uric-acid–induced inflammation, of *Eucommia ulmoides* leaf ingredients. The disease represents the common gene of hyperuricemia, gouty arthritis, and urinary nephritis

By screening the Swiss Target Prediction and SEA databases for the targets of the 32 compounds, 669 potential targets related to hyperuricemia, gouty arthritis, and UA-induced nephritis were identified. Likewise, GeneCards and OMIM yielded 2039 potential targets. Wayne analysis revealed 219 overlapping targets between the compound targets and disease targets. These 219 targets were defined as the common targets related to hyperuricemia and UA-induced inflammation of *E. ulmoides* iridoids and flavonoids (Figure 1).

***Composition–candidate-target interaction network***

**Figure 2:**
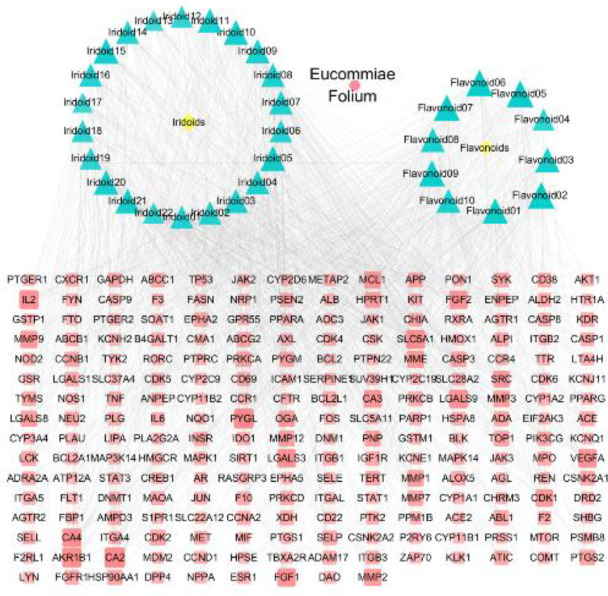
Composition of the target network

By using Cytoscape 3.6.0, the effects of *E. ulmoides* iridoids and flavonoids were mined. Consequently, 219 candidate targets of the 32 compounds were obtained. Then, a component–target network diagram, comprising 256 nodes and 1576 edges, was constructed. This diagram was adjusted according to the degree values. A high degree value was indicated by a large shape and dark color (Figure 2). The main core components were astragalin (56), isoquercetin (56), kaempferol (55), hirsutin (54), quercetin 3-O-sambubioside (53), quercetin (51), 7-epi-loganin (51), harpagide acetate (51), and (2S,3S)-taxifolin-3-O-β--glucopyranoside (49), among which eight were flavonoids and two were iridoids. These compounds were considered *E. ulmoides* leaf ingredients that may be used for the treatment of hyperuricemia and UA-induced inflammation.

***Construction and analysis of the PPI network diagram***

**Figure 3:**
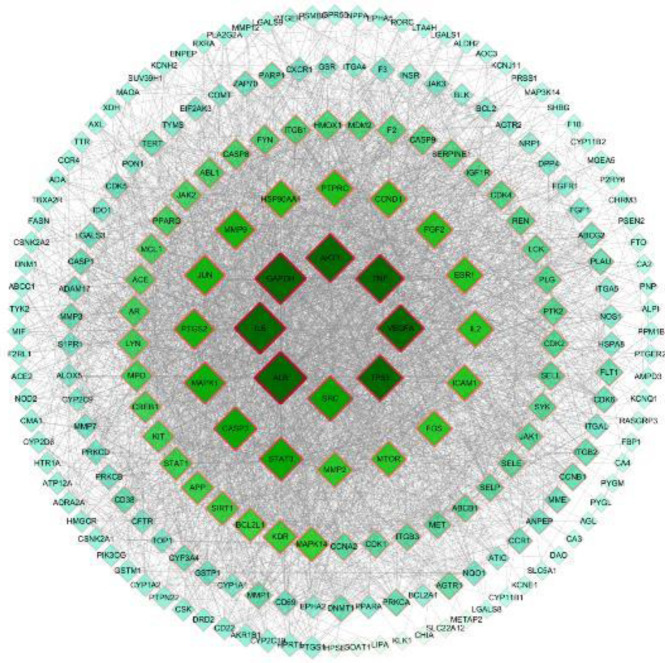
PPI network of *Eucommia ulmoides *leaf ingredients for the treatment of hyperuricemia and uric-acid-induced inflammation

By using the STRING database, we constructed the PPI network diagram, in which the targets separated from the other protein networks were deleted. The differently colored lines in the figure correspond to different relationship sources, and the determined interaction relationships are indicated in light blue and rose red. The lines were connected, the light blue was selected from the database, and the rose-red was experimentally determined. The predicted interaction relationship was connected by the green and thick red and purple lines. Cytoscape 3.6.0 was used to perform network analysis on the PPI results obtained from the STRING database, and 83 core targets with values higher than the average degree value (32.95) and average proximity centrality value (0.503887116) were obtained. The top 10 targets are shown in Table 2. The degree value referred to the number of connections between a network node and other nodes, the proximity centrality value is a measure of the importance of a node according to the transfer distance between nodes, and both values can be used to determine whether a target protein is a “key target”. The color depth, shape, and edge thickness of the PPI map were adjusted according to the degree values. A dark color meant a high degree value, and a large shape meant a thick edge (Figure 3). 

***Target GO function annotation***

After constructing the PPI network, 83 core targets with correlation values higher than the average value were imported into the WebGestalt database for GO enrichment analysis. Via GO functional annotation, 12 target genes were classified into “biological process” (BP), 19 into “cell component” (CC), and 16 into “molecular function” (MF). The genes under the BP category were primarily related to biological regulation, response to stimuli, and metabolic processes. Membrane, nucleus, and protein-containing complexes accounted for a large proportion of the CC-related genes. Protein binding, ion binding, and transferase activity had the greatest impact on the MF-related genes (Figure 4). *E. ulmoides* leaf components were found to regulate 390 BPs related to hyperuricemia and UA-induced inflammation, and these BPs primarily involved UA metabolism, lipid metabolism, inflammation, and immune function. Those for UA metabolism were as follows: positive regulation of defense response (GO:0031349), reaction to compounds containing purines (GO:0014074), negative regulation of transferase activity (GO:0051348), and negative regulation of catabolic processes (GO: 0009895). Those for lipid metabolism were as follows: regulation of lipid metabolism (GO:0019216), adipocyte differentiation (GO:0045444), response to lipoprotein particles (GO:0055094), cell response stimulated by lipoprotein particles (GO:0071402), and regulation of lipase activity (GO:0060191). Those for inflammatory response were as follows: oxidative stress response (GO:0006979), response to antibiotics (GO:0046677), regulation of inflammatory response (GO:0050727), response to acidic chemicals (GO:0001101), and interleukin-6 (GO:0070741). Those for immune function were as follows: immune response regulation signaling pathway (GO:0002764), neutrophil-mediated immunity (GO:0002446), adaptive immune response (GO:0002250), regulation of innate immune response (GO:0045088), regulation of immune effect processes (GO:0002697), and production of immune response molecular mediators (GO:0002440). These four main aspects explain the complex multi-path effect of *E. ulmoides* leaf components in ameliorating hyperuricemia.

***KEGG pathway analysis***

**Figure 5:**
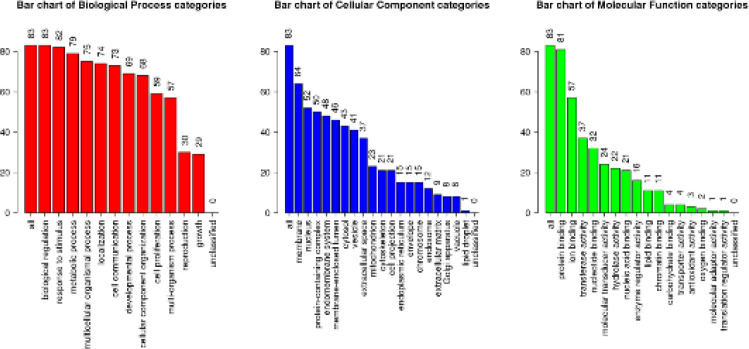
KEGG enrichment analysis

The KEGG Orthology Based Annotation System 3.0 (KOBAS 3.0) database was used to perform KEGG pathway enrichment analysis on the core targets, and 201 KEGG pathways with *P*<0.05 were obtained. The first 20 pathways were selected as high-level bubble graphs for visual display (Figure 5). These pathways included the PI3K-AKT signaling pathway (hsa04151), AGE-RAGE signaling pathway in diabetic complications (hsa04933), endocrine-resistance signaling pathway (hsa01522), and fluid shear stress and atherosclerosis signaling pathway (hsa05418). Uric acidemia and UA-induced inflammation were closely related.

***Molecular-docking results***

The 32 compounds selected from *E. ulmoides* leaf ingredients, and 5 drugs used for the treatment of UA-induced inflammation or hyperuricemia (allopurinol, benzbromarone, probenecid, febuxostat, and colchicine), a total of 37 compounds and proteins including UA production, reabsorption, transport xanthine oxidoreductase (XO), glucose transporter 9 (GLUT9), organic anion transporter 1 (OAT1), organic anion transporter 3 (OAT3), organic cation transporter 1 (OCT1), and ATP-binding cassette subfamily G member 2 (ABCG2), and NLRP3/ASC/Caspase-1 signal axis related proteins: NLRP3, apoptosis-associated speck-like protein containing a caspase activation and recruitment domain (ASC), Procaspase-1. Caspase-1(CASP1), nuclear factor of kappa light polypeptide gene enhancer in B-cell 1 (NFKB1), myeloid differentiation primary response protein 88 (MyD88), Toll-like receptor4 (TLR4), and mitogen-activated protein kinase 8 (MAPK8) were docked. The molecular-docking results showed that the flavonoids kaempferide-3-O-β--glucopyranoside and nicotiflorin did not bind to any of the proteins. A total of 39 docked compounds were compatible with Procaspase-1 (3E4C), OAT1 (6VO5), XO (1FIQ), ASC (5TM4), GLUT9 (5EQG), TLR4 (3ULA), and MAPK8 (4HYU). These compounds all had different degrees of binding, and most of the compounds were bound to OAT3 (3AT3), indicating that they were *E. ulmoides* leaf flavones. The combination of the iridoid components with UA production, excretion, reabsorption, and inflammation-related proteins up-regulated by high serum UA levels revealed that these components may inhibit UA production, promote UA excretion, and reduce inflammation (Figure 6).

The flavonoids rutin, kaemphferol-3-O-β--rutinoside, astragalin, (2S,3S)-taxifolin-3-O-β-- glucopyranoside, isoquercetin, kaempferol, and quercetin, and the iridoids eucomoside B, eucomoside C, deacetylasperulosidic acid, daphylloside, asperuloside acid, and 8-epi-loganin bound to XO, which regulates UA production more than its inhibitor febuxostat (-59.94). Among these compounds, rutin (-87.92) and kaemphferol-3-O-β--rutinoside (-86.52) had the strongest binding forces. The flavonoids quercetin, isoquercetin, (2S,3S)-taxifolin-3-O-β--glucopyranoside, astragalin, and quercetin 3-O-sambubioside, the iridoids eucomoside C and ulmoside C, and the inflammation-related regulatory proteins Procaspase-1 (3E4C), ASC (5TM4), TLR4 (3ULA), and MAPK8 (4HYU) had stronger binding capacities than colchicine, which has anti-inflammatory effects. Most of the docked compounds had strong binding forces to the UA-excretion–related proteins GLUT9 (5EQG), OAT1 (6VO5), and OAT3 (3AT3).

Results of cluster analysis showed that allopurine and loliolide were clustered together, and febuxostat, probenecid, quercetin, (2S,3S)-taxifolin-3-O-β--glucopyranoside, and kaempferol were segregated into a different cluster than that of allopurine and loliolide. Benzbromarone and aucubin were grouped together, and colchicine was grouped with isoquercetin, eucomoside B, eucomoside C, and daphylloside (Figure 6). The active ingredients isoquercetin, kaempferol, and quercetin had strong binding forces and were clustered with positive drugs. These ingredients were also potential active ingredients predicted through network pharmacology as they were docked with XO, GLUT9, OAT1, ASC, TLR4, or MAPK8 proteins (Figure S1).

***Effects of Eucommia ulmoides leaf extract on renal index and renal function in rats on HFFD***

To further determine our predicted results from network pharmacology and molecular docking, we used Wistar rats as an *in vivo* model of experimental hyperuricemia and kidney injury caused by HFFD. The renal index and renal function of the rats on HFFD were significantly changed at the end of the treatment period. Renal index and serum levels of UA, CRE, and BUN were significantly higher in the HFFD group than in the CON group (*P*<0.05, *P*<0.01) (Figure 7), were not significantly different between CON+EULH (200 mg/kg) and CON groups (*P*>0.05), and were significantly higher in HFFD+EULL (100 mg/kg), HFFD+EULH (200 mg/kg), and HFFD+MF (100 mg/kg) groups than in the HFFD group (*P*<0.01, *P*<0.05).

***Effects of the Eucommia ulmoides leaf extract on serum TNF-α and IL-6 levels in rats on HFFD***

The network pharmacology prediction of *E. ulmoides* leaf components to hyperuricemia and uric nephritis showed that IL-6 (2) and TNF (5) were among the top 10 core targets. To verify the anti-inflammatory activity of the *E. ulmoides* leaf extract, the serum levels of the inflammatory factors TNF-α and IL-6 in the treated rats were measured. These levels were significantly higher in the HFFD group than in the CON group (*P*<0.01) (Figure 8), were not significantly different between CON+EULH (200 mg/kg) and CON groups (*P*>0.05), and were significantly higher in HFFD+EULL (100 mg/kg), HFFD+EULH (200 mg/kg) , and HFFD+MF (100 mg/kg) groups than in the HFFD group (*P*<0.01, *P*<0.05).

***Effect of Eucommia ulmoides leaf extract on renal pathological changes in rats on HFFD***

Since an elevated serum UA level leads to acute kidney injury and chronic kidney disease, we explored the effect of *E. ulmoides* leaf extract on the renal pathological changes in rats on HFFD by HE staining of kidney sections. The results showed that, compared with the CON group, the HFFD group showed serious histopathological damage, mainly glomerular enlargement, glomerular adhesion, and narrowing or even disappearance of the glomerular cavity (Figure 9). Compared with the HFFD group, HFFD+EULL (100 mg/kg), HFFD+EULH (200 mg/kg), and HFFD+MF (100 mg/kg) groups had significantly reduced glomerulomegaly and glomerular cystic stenosis. These results further suggest that long-term intake of high-fat/high-fructose diets can induce kidney injury and impair kidney function and that the intervention with *E. ulmoides* leaf extract can effectively prevent this injury and maintain a healthy kidney function.

***Effects of Eucommia ulmoides leaf extract on TLR4 and GLUT9 protein levels in the kidney***

To investigate the mechanism of action of the *E. ulmoides *leaf extract in preventing serum UA level increase and kidney injury induced by HFFD in rats and to verify the predicted results of molecular docking, we measured the levels of TLR4 and GLUT9 proteins in the kidneys via immunofluorescence assay. Immunofluorescence results showed that (Figure 10), compared with the levels in the CON group, the levels of TLR4 and GLUT9 proteins in the HFFD group were significantly increased (*P*<0.01), and no significant difference was between CON+EULH (200 mg/kg) and CON groups (*P*>0.05). Compared with the level in the HFFD group, the TLR4 levels in HFFD+EULL (100 mg/kg), HFFD+EULH (200 mg/kg), and HFFD+MF (100 mg/kg) groups were significantly reduced (*P*<0.01). Compared with the level in HFFD group, the GLUT9 levels in HFFD+EULH (200 mg/kg) and HFFD+MF (100 mg/kg) groups were significantly reduced (*P*<0.01).

**Table 1:** Iridoids and flavonoids in *Eucommia ulmoides * leaves

Number	Ingredient name	Number	Ingredient name
Flavonoid01	Quercetin	Iridoid07	Reptoside
Flavonoid02	Isoquercetin	Iridoid08	Aucubigenin
Flavonoid03	(2S,3S)-taxifolin-3-O-β-D-glucopyranoside	Iridoid09	Eucomoside A
Flavonoid04	Thunberginol C	Iridoid10	Eucomoside B
Flavonoid05	Astragalin	Iridoid11	Eucomoside C
Flavonoid06	Kaempferol	Iridoid12	Daphylloside
Flavonoid07	Hirsutin	Iridoid13	Scandoside methyl ester
Flavonoid08	Nicotiflorin	Iridoid14	Loganin
Flavonoid09	Quercetin 3-O-sambubioside	Iridoid15	7-epi-loganin
Flavonoid10	Rutin	Iridoid16	8-epi-loganin
Iridoid01	Deacetylasperulosidic acid	Iridoid17	Deacetyl asperulosidic acid methyl ester
Iridoid02	Aucubin	Iridoid18	Ulmoside C
Iridoid03	Geniposide	Iridoid19	Ulmoside D
Iridoid04	Geniposidic acid	Iridoid20	Loliolide
Iridoid05	Harpagide acetate	Iridoid21	Asperuloside
Iridoid06	Ajugoside	Iridoid22	Asperuloside acid

KEGG: Kyoto Encyclopedia of Genes and Genomes

**Figure 4:**
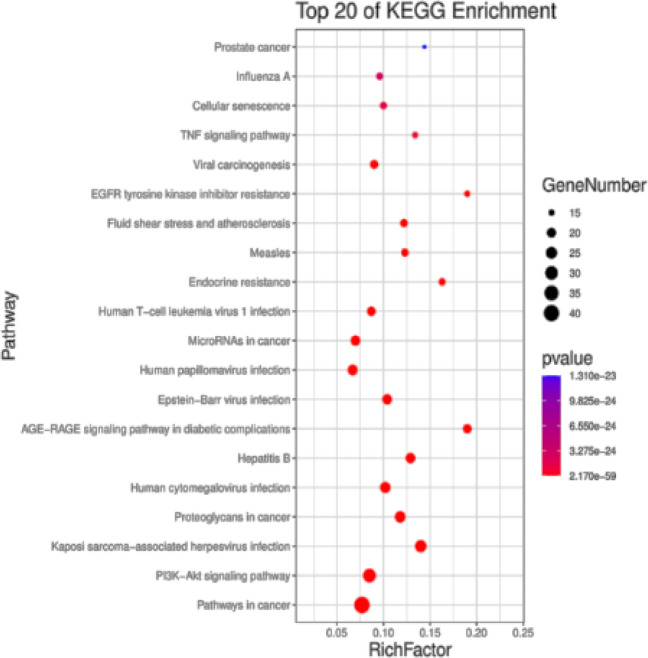
GO enrichment analysis

**Figure 6.**
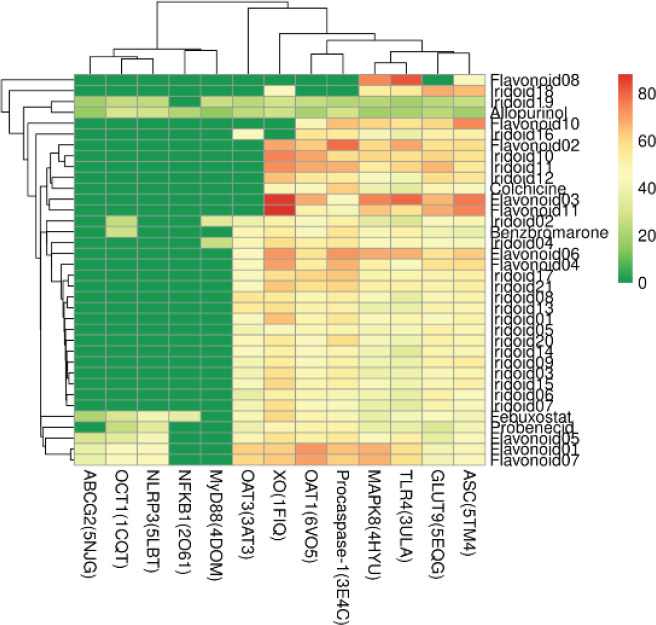
Molecular-docking CDOCKER-INTERACTION-ENERGY heat map

**Figure 7:**
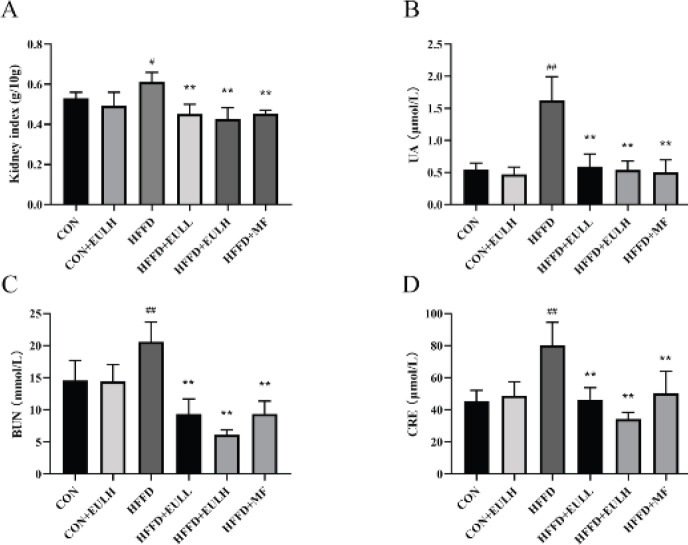
Effects of *Eucommia ulmoides* leaf extract on renal index and serum levels of UA, CRE, and BUN. A) Renal index of the rats in each experimental group. B) Serum UA level of the rats in each experimental group. C) Serum CRE level of the rats in each experimental group. D) Serum BUN level of the rats in each experimental group. The data are the mean ± SD of each group, n = 6.** P<0.01, compared with CON group; #* P*<0.05 and ##* P*<0.01, compared with HFFD group

UA: Uric acid; HFFD: high-fat and high-fructose diet

**Figure 8:**
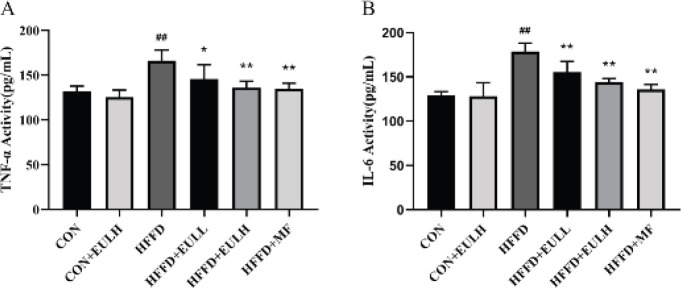
Effects of *Eucommia ulmoides* leaf extract on serum levels of the inflammatory factors TNF-α and IL-6. A) The serum TNF-α level in each experimental group. B) The serum IL-6 level in each experimental group. The data are the mean ± SD of each group, n = 6. *P<0.05, **P<0.01, compared with CON group; ##*P*<0.01, compared with HFFD group

HFFD: high-fat and high-fructose diet

**Figure 9:**
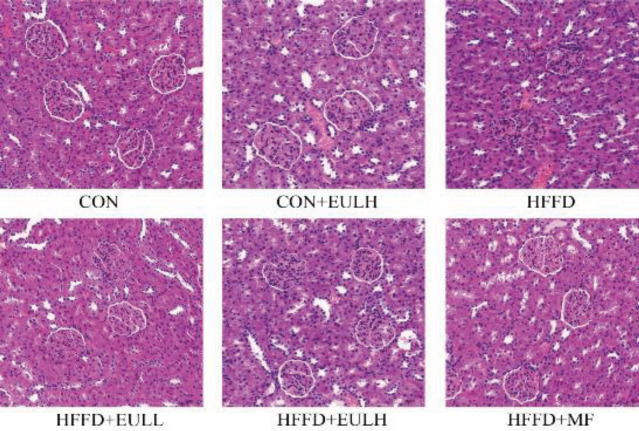
HE staining to evaluate the effect of the *Eucommia ulmoides* leaf extract on the renal pathological changes in rats on a high fat/high fructose diet

**Figure 10:**
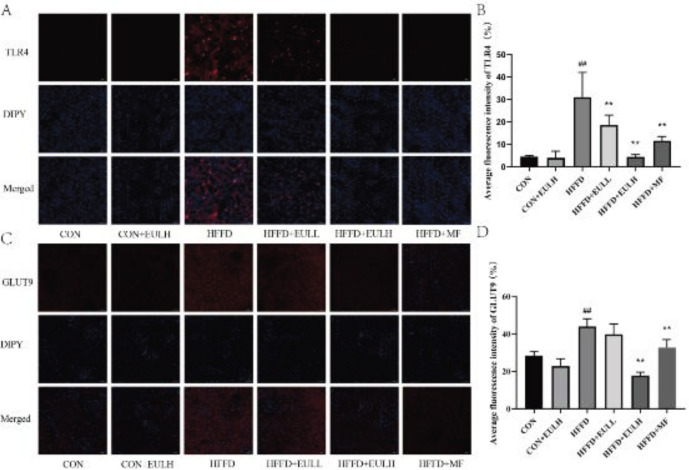
Effects of *Eucommia ulmoides* leaf extract on the levels of TLR4 and GLUT9 proteins in rat kidney tissues were detected via immunofluorescence analysis. A) Expression of TLR4 in each experimental group. B) TLR4 average fluorescence intensity in each experimental group. C) Expression of GLUT9 in each experimental group. D) GLUT9 average fluorescence intensity in each experimental group. The data are the mean ± SD of each group, n = 6.**P<0.01, compared with the CON group; ##*P*<0.01, compared with the HFFD group

HFFD: high-fat and high-fructose diet

## Discussion

Hyperuricemia (serum UA level > 6 mg/dl) is currently one of the most common metabolic diseases. It is caused by excessive serum lipid levels or reduced renal excretion (16). High serum UA level leads to deposition of urate crystals in the joints and kidneys, which can induce inflammation (e.g., gouty arthritis and kidney stones) and accelerate the progression of chronic kidney disease, obesity, atherosclerotic heart disease (17, 18). At present, the drugs used for the treatment of hyperuricemia and UA-induced inflammation are primarily anti-inflammatory drugs and those that control the level of UA in the body, such as colchicine, allopurine, and benzbromarone. However, these drugs are known to cause severe gastrointestinal irritation. Severe adverse reactions, such as bone marrow suppression and nephrotoxicity, limit their clinical application (19). Many studies have shown that flavonoids have antiuricemic and anti-inflammatory activities (20). *In vitro*, flavonoids, phenols, iridoid glycosides, and coumarins have anti-gout effects through inhibition of XO (21). 

In the present study, by using a network pharmacology approach, we systematically investigated the molecular target networks of the major classes of *E. ulmoides* leaf ingredients for prevention and treatment of hyperuricemia and related diseases. There were 219 intersecting targets between 32 components (22 Iridoids and 10 Flavonoids) of *E. ulmoides* leaves and several diseases, such as hyperuricemia, gouty arthritis, and uratic nephritis. Among these targets, albumin (ALB), Interleukin-6(IL6), glyceraldehyde-3-phosphate dehydrogenase (GAPDH), AKT1, tumor necrosis factor (TNF), vascular endothelial growth factor A (VEGFA), tumor antigen gene p53(TP53), proto-oncogene tyrosine-protein kinase SRC (SRC), caspase-3 (CASP3), and signal transducers and activators of transcription3 (STAT3) were the core targets of the *E. ulmoides* leaf extract to suppress hyperuricemia and UA-induced inflammation. The 390 biological processes enriched among the *E. ulmoides* leaf components to suppress hyperuricemia and UA-induced inflammation are mainly involved in UA metabolism, lipid metabolism, inflammation, and immune function. Enrichment analysis showed that these targets are involved in various hyperuricemia-related pathways, such as the PI3K-AKT signaling pathway (hsa04151) (22), AGE-RAGE signaling pathway in diabetic complications (hsa04933), endocrine-resistance signaling pathway (hsa01522), fluid shear stress and atherosclerosis signaling pathway (hsa05418), and other pathways related to hyperuricemia and UA-induced inflammation. Animal experiments confirmed that the targets IL6 and TNF are involved in elevation of serum UA levels and inflammatory response induced by HFFD. Furthermore, the *E. ulmoides* leaf extract could lower serum UA levels and suppress kidney inflammation and UA reabsorption, and reverse kidney damage, and thus this extract emerges as a very promising drug for the treatment of hyperuricemia.

Notably, hyperuricemia can induce renal inflammation through crystal-dependent and crystal-independent pathways. The study by Braga *et al.* on the pathogenic effect of UA points out that the inflammatory response caused by high UA levels is the main mechanism underlying gout (5). Monosodium urate (MSU) crystals can induce an inflammatory response, which is recognized by toll-like receptor (TLR)-2 and TLR-4 (23). MSU also triggers neutrophil activation and promotes the production of immune mediators, resulting in an inflammatory response (24). There is growing evidence that asymptomatic hyperuricemia may lead to diseases such as hypertension, obesity, diabetes, and chronic kidney disease by stimulating inflammation (25). TLR4/ MyD88 signaling is activated in the kidney of fructose-fed rats, subsequently leading to activation of the nuclear factor-κB (NF-κB) signaling and resulting in inflammatory responses (26, 27). The results of the present study showed that the *E. ulmoides* leaf extract down-regulated renal expression of TLR4, a protein related to kidney inflammation, and thereby showed anti-inflammatory activity in rats on HFFD.

Serum UA level is regulated by UA transport proteins in the kidney and intestine, specifically GLUT9 (SLC2A9), urate transporter-1(URAT1) (SLC22A12), and ATP-binding cassette transporter G2 (ABCG2) (28). GLUT9, encoded by the SLC2A9 gene, is an important proximal tubular transporter protein for UA and plays a key role in hyperuricemia. Thus, it is considered an important target for drug therapy (29, 30). In this study, we showed that the *E. ulmoides* leaf extract could suppress the elevation of serum UA level induced by HFFD, and the molecular docking prediction results showed that all the major classes of components in *E. ulmoides* leaves could bind to GLUT9, which was verified through immunofluorescence analysis. The results showed that the *E. ulmoides* leaf extract could reduce renal GLUT9 protein levels and inhibit the reabsorption of UA in the kidney.

## Conclusion

In this study, a network of *E. ulmoides* leaf components and hyperuricemia-related diseases was constructed, and the *E. ulmoides* leaf components were found to be significantly enriched in various inflammation-related pathways. Molecular docking results showed that cyclic enol ether terpenes and flavonoids are likely to bind to proteins related to inflammation and UA metabolism. In addition, the effects of the *E. ulmoides* leaf extract on the candidate targets IL-6, TNF-α, TRL4, and GLUT9 were verified via animal experiments. These results highlight that the *E. ulmoides* leaf extract modulates UA levels, prevents kidney injury and inflammation, and provides a theoretical basis for developing therapeutics based on the bioactive components of this extract.

## Authors’ Contributions

MG, LPD, and ZMW Conceived and designed the study. MG, QXL, and WJZ Performed research. XQL, HZ, NH, and YZ Analyzed data. MG, AYC, and HZ Wrote the paper. All authors affirmed the final manuscript before submitting.

## Conflicts of Interest

The author declares that they have no competing interests associated with the manuscript.

## References

[1] Caliceti C, Calabria D, Roda A, Cicero AFG (2017). Fructose Intake, serum uric acid, and cardiometabolic disorders: A critical review. Nutrients.

[2] Nakagawa T, Tuttle KR, Short RA, Johnson RJ (2005). Hypothesis: Fructose-induced hyperuricemia as a causal mechanism for the epidemic of the metabolic syndrome. Nat Clin Pract Nephrol.

[3] Jia G, Habibi J, Bostick BP, Ma L, DeMarco VG, Aroor AR (2015). Uric acid promotes left ventricular diastolic dysfunction in mice fed a Western diet. Hypertension.

[4] Stack AG, Hanley A, Casserly LF, Cronin CJ, Abdalla AA, Kiernan TJ (2013). Independent and conjoint associations of gout and hyperuricaemia with total and cardiovascular mortality. Qjm.

[5] Braga TT, Foresto-Neto O, Camara NOS (2020). The role of uric acid in inflammasome-mediated kidney injury. Curr Opin Nephrol Hypertens.

[6] Zhang S, Wang Y, Cheng J, Huangfu N, Zhao R, Xu Z (2019). Hyperuricemia and cardiovascular disease. Curr Pharm Des.

[7] Liu E, Han L, Wang J, He W, Shang H, Gao X (2012). Eucommia ulmoides bark protects against renal injury in cadmium-challenged rats. J Med Food.

[8] Lee G, Lee H, Choi M, Choi A, Shin T, Chae H (2018). Eucommia ulmoides leaf (EUL) extract enhances NO production in ox-LDL-treated human endothelial cells. Biomed Pharmacother.

[9] Zhang Y, Zhang H, Wang F, Yang D, Ding K, Fan J (2015). The ethanol extract of Eucommia ulmoides Oli leaves inhibits disaccharidase and glucose transport in Caco-2 cells. J Ethnopharmacol.

[10] Hao S, Xiao Y, Lin Y, Mo Z, Chen Y, Peng X (2016). Chlorogenic acid-enriched extract from Eucommia ulmoides leaves inhibits hepatic lipid accumulation through regulation of cholesterol metabolism in HepG2 cells. Pharm Biol.

[11] Lee G, Lee H, Park S, Shin T, Chae H (2019). Eucommia ulmoides leaf extract ameliorates steatosis induced by high-fat diet in rats by increasing lysosomal function. Nutrients.

[12] Wang C, Tang L, He J, Li J, Wang Y (2019). Ethnobotany, phytochemistry and pharmacological properties of Eucommia ulmoides: A review. Am J Chin Med.

[13] Fang C, Chen L, He M, Luo Y, Zhou M, Zhang N (2019). Molecular mechanistic insight into the anti-hyperuricemic effect of Eucommia ulmoides in mice and rats. Pharm Biol.

[14] Suganthy N, Devi K, Nabavi S, Braidy N, Nabavi S (2016). Bioactive effects of quercetin in the central nervous system: Focusing on the mechanisms of actions. Biomed Pharmacother.

[15] Hosoo S, Koyama M, Watanabe A, Ishida R, Hirata T, Yamaguchi Y (2017). Preventive effect of Eucommia leaf extract on aortic media hypertrophy in Wistar-Kyoto rats fed a high-fat diet. Hypertens Res.

[16] Garofalo C, De Stefano T, Vita C, Vinci G, Balia F, Nettuno F (2018). [Hyperuricaemia and Chronic Kidney Disease]. G Ital Nefrol.

[17] Landolfo M, Borghi C (2019). Hyperuricaemia and vascular risk: the debate continues. Curr Opin Cardiol.

[18] Borghi C, Agabiti-Rosei E, Johnson RJ, Kielstein JT, Lurbe E, Mancia G (2020). Hyperuricaemia and gout in cardiovascular, metabolic and kidney disease. Eur J Intern Med.

[19] Shekelle P, Newberry S, FitzGerald J, Motala A, O’Hanlon C, Tariq A (2017). Management of gout: A systematic review in support of an american college of physicians clinical practice guideline. Ann Intern Med.

[20] Masuda T, Shingai Y, Takahashi C, Inai M, Miura Y, Honda S (2014). Identification of a potent xanthine oxidase inhibitor from oxidation of caffeic acid. Free Radic Biol Med.

[21] Ling X, Bochu W (2014). A review of phytotherapy of gout: Perspective of new pharmacological treatments. Pharmazie.

[22] Liu L, Zhao T, Shan L, Cao L, Zhu X, Xue Y (2021). Estradiol regulates intestinal ABCG2 to promote urate excretion via the PI3K/Akt pathway. Nutr Metab (Lond).

[23] So A (2008). Developments in the scientific and clinical understanding of gout. Arthritis Res Ther.

[24] Jin M, Yang F, Yang I, Yin Y, Luo JJ, Wang H (2012). Uric acid, hyperuricemia and vascular diseases. Front Biosci (Landmark Ed).

[25] Joosten LAB, Crişan TO, Bjornstad P, Johnson RJ (2020). Asymptomatic hyperuricaemia: A silent activator of the innate immune system. Nat Rev Rheumatol.

[26] Tan J, Wan L, Chen X, Li X, Hao X, Li X (2019). Conjugated linoleic acid ameliorates high fructose-induced hyperuricemia and renal inflammation in rats via NLRP3 inflammasome and TLR4 signaling pathway. Mol Nutr Food Res.

[27] Yang Y, Zhang DM, Liu JH, Hu LS, Xue QC, Ding XQ (2015). Wuling san protects kidney dysfunction by inhibiting renal TLR4/MyD88 signaling and NLRP3 inflammasome activation in high fructose-induced hyperuricemic mice. J Ethnopharmacol.

[28] Scuiller A, Pascart T, Bernard A, Oehler E (2020). [Gout]. Rev Med Interne.

[29] Wang M, Zhao J, Zhang N, Chen J (2016). Astilbin improves potassium oxonate-induced hyperuricemia and kidney injury through regulating oxidative stress and inflammation response in mice. Biomed Pharmacother.

[30] Zhou Y, Zhang X, Li C, Yuan X, Han L, Li Z (2018). Research on the pharmacodynamics and mechanism of fraxini cortex on hyperuricemia based on the regulation of URAT1 and GLUT9. Biomed Pharmacother.

